# Exposing the Risk: Identifying Perspectives on Skin Cancer Awareness and Prevention Opportunities Among Hispanic Outdoor Workers

**DOI:** 10.1007/s40615-025-02623-2

**Published:** 2025-09-05

**Authors:** David Perez, Dariana Sedeño-Delgado, Carlos Orellana García, Valeria Gómez, Chiranjeev Dash, Roxanne Mirabal-Beltran, Alejandra Hurtado-de-Mendoza

**Affiliations:** 1 Georgetown University Medical Center, Washington, DC, USA; 2 Baylor College of Medicine, Houston, TX, USA; 3 Georgetown University Berkley School of Nursing, Washington, DC, USA; 4 Jess and Mildred Fisher Center for Hereditary and Clinical Cancer Genomics Research, Washington, DC, USA; 5 Lombardi Comprehensive Cancer Center, Georgetown University, 2115 Wisconsin AVE NW, Suite 300, Washington, DC 2000, USA

**Keywords:** Skin cancer prevention, Hispanic outdoor workers, Occupational health, Skin cancer, Mixed-methods

## Abstract

**Introduction:**

Hispanics are over-represented in outdoor occupations; therefore they face an elevated risk of skin cancer. However, there is limited research examining these workers’ self-risk perceptions and sun-protective behaviors. This study explores Hispanic outdoor workers’ knowledge, attitudes, barriers, and facilitators for engaging in sun-protective behaviors to inform a culturally-tailored intervention.

**Methods:**

We conducted a mixed methods study with Hispanic outdoor workers (*n* = 25) over 18 years old. Three focus groups were held in-person or virtually. Participants were recruited through community partners, flyers, and referrals. Focus groups were conducted in Spanish, recorded, transcribed, and analyzed using Rapid Qualitative Analysis. We used descriptive statistics to analyze quantitative data.

**Results:**

Most participants were male (88%), 40% completed up to 8th grade education, and 60% were uninsured. Results revealed four key themes: skin cancer awareness, risk perception, risk-reduction strategies, and barriers/facilitators for risk-reduction behaviors. Participants reported daily sun exposure ranging from 3 to 10 h, often with limited access to shade. Sunglasses (57.1%) and long sleeves (52.4%) were commonly used, while sunscreen use remained low (19.0%) due to discomfort, stigma, and limited knowledge. Many participants (20%) never took protective measures and expressed uncertainty about their skin cancer risk (40%). Despite these challenges, participants showed significant interest in learning more about sun protection.

**Discussion:**

Our findings suggest that Hispanic outdoor workers have limited knowledge and low perceived skin cancer risk. This study highlights the need for tailored educational initiatives to address skin cancer prevention for Hispanic outdoor workers.

## Introduction

Skin cancer is the most frequently diagnosed cancer in the United States, with over 5 million new cases reported annually [1]. In 2025, projections indicate that approximately 5.4 million cases of non-melanoma skin cancer (basal cell carcinoma (BCC) and squamous cell carcinoma (SCC)) and 104,960 cases of melanoma will be diagnosed [1, 2]. The most common modifiable risk factor for skin cancer is exposure to solar ultraviolet (UV) radiation, making skin cancer highly preventable [3–7].

Outdoor workers are exposed to a high-risk environment of long periods of sun-exposure, receiving up to 8 times more UV radiation than indoor workers and facing a 60% greater risk of developing skin cancer as a result [8]. UV radiation can cause DNA damage within skin cells, and this damage can accumulate over time, causing mutations that lead to uncontrolled cell growth, resulting in skin cancer [9]. Hispanics represent a majority of outdoor workers, and are overrepresented in construction, landscaping, and agricultural sectors [10–12]. The incidence of both non-melanoma and melanoma skin cancers has risen in Hispanics over the past decades [13–15]. Despite a lower incidence of skin cancer compared to non-Hispanic whites, Hispanics have poorer prognoses and cancer outcomes [16]. Hispanics are more likely to be diagnosed at later stages of skin cancer, when the cancer has progressed significantly, leading to lower survival rates and poorer outcomes. This disparity is particularly evident in cases of melanoma–the deadliest form of skin cancer–and non-melanoma skin cancers (BCC and SCC) [17].

Primary prevention strategies such as wearing sunscreen, seeking shade, and wearing protective clothing have been shown to significantly reduce UV exposure and the associated risk of skin cancer [18–22]. Secondary prevention, including regular self-skin examinations and provider-performed total body skin exams, facilitates early detection, which is critical for improving outcomes [18–22]. Studies have consistently shown that Hispanics have very low engagement in primary skin cancer prevention and secondary skin cancer prevention behaviors [18–22]. The few studies that have focused on Hispanic outdoor workers have also found suboptimal sun protection behaviors, despite high exposure to UV radiation [23–26].

While there are established recommendations and resources informing the public about the dangers of sun exposure and the risk of skin cancer, most interventions and public health campaigns have been targeted to NHW populations [27–29]. Cultural beliefs and social norms often influence health decision-making, and for many Hispanics, the understanding of sun exposure risks may not align with conventional prevention strategies [30]. These cultural factors, along with others such as limited knowledge and awareness, low health literacy and numeracy, limited access to healthcare, language barriers, and competing priorities, further complicate efforts to promote effective sun protection within this population [31].

Few studies have reported on skin cancer knowledge and risk perception in Hispanic outdoor workers [23–26], but gaps still exist in assessing outdoor workers’ understanding of topics such as sun exposure, skin cancer risk perception, protective measures, and barriers and facilitators to engage in protective behaviors. Research regarding industry-specific variations in sun exposure and protection also remains limited, highlighting the need to expand research across a larger range of Hispanic outdoor workers, including those in agriculture, construction, and landscaping.

We conducted a mixed methods study to understand Hispanic outdoor workers’ awareness of skin cancer and barriers and facilitators to engage in protective behaviors to inform the development of culturally-tailored skin cancer prevention intervention for this population.

## Methods

Using a mixed methods study design with simultaneous collection of qualitative and quantitative data, we conducted three focus groups in-person and virtually between October and November 2024 [32]. This approach aligns with the mixed methods framework outlined by Palinkas et al. [32] which supports the concurrent collection and analysis of qualitative and quantitative data to provide a more comprehensive understanding of the factors being studied. Qualitative data were prioritized as the primary source of insight, while quantitative data were used to contextualize findings. As Palinkas et al. [32] note, the strength of this method lies in its ability to combine the depth of qualitative insights with the applicability of quantitative data, allowing for triangulation and validation of the findings.

Participants were recruited through community outreach methods, including community partners who provide education to outdoor workers, as well as through flyers and referrals to the study. To be eligible, participants had to self-identify as Hispanic, be 18 years of age or older, work outdoors or have experience working outdoors within the industries of agriculture, landscaping, and/or construction.

Each focus group had between six to ten participants (n = 25). Prior to initiating focus group discussions, participants provided verbal consent to participate in the study and be audio taped. Afterward, they completed a short survey to assess sociodemographic and psychosocial factors. Focus groups lasted approximately two hours, in-person or remotely, and were conducted in Spanish with a bilingual moderator.

### Measures

#### Sociodemographic Factors

The sociodemographic information collected included race, ethnicity, gender, country of birth, marital status, level of education, annual income, health insurance status, and employment status.

#### Psychosocial Factors

Sun exposure: We asked participants to report the range of hours they work under direct sunlight on any given day to assess sun exposure [33]. Sun Protection Practices: Four different survey items assessed current sun protection practices. The first item assessed use of sun protection during 1 h or more of labor under the sun, with “Yes”, “No”, and “I don’t know” responses. A follow-up question asked participants to select all methods of sun protection they currently use [33]. Response options included: sunscreen, hat, sunglasses, long sleeves/long pants, or other (e.g., bandana). Using two items from NIH’s HINTS, we assessed how often participants protect their skin from sun exposure during their workday [34] and stay in the shade [34] on a 5-point Likert type scale. Responses to these questions ranged on a scale from “Never” to “Always”. Perceived Risk: Two items from NIH’s HINTS were used to assess perceived risk. The first item asked participants to describe their perceived risk of skin cancer compared to someone their age. Response options included “More likely,” “Equally Likely,” “Less Likely,” “I don’t Know,” and “Prefer not to Answer.” [34]. The second question asked about their perceived chances of developing skin cancer in the future on a 5-point Likert-type scale. Response options ranged from “Very Unlikely” to “Very Likely” [34]. Cancer Worry was assessed with an item from HINTS [34] asking how often they worry about getting skin cancer on a 5 point Likert-type scale. Response options ranged from “Never” to “Always” [34].

### Focus Group Guide

The focus groups aimed to gather information on the participants’pre-existing knowledge and awareness of skin cancer, specifically focusing on their understanding of general skin cancer knowledge, risk perception, and health messaging. Our dialogue with focus group members began with open-ended questions about participants’ work lives (e.g., “What is a typical workday like for you?”), followed by prompts to assess their baseline knowledge of skin cancer and perceived risk (e.g., “What do you know about skin cancer?”, “Do you feel that the risk of skin cancer is relevant to you?”). Subsequent questions focused on specific risk-reducing practices, such as sunscreen use, access to shade, and the use of sun-protective clothing. Lastly, we concluded the sessions by asking about suggestions to develop a culturally-tailored video about skin cancer prevention and strategies to disseminate the video.

We chose a focus group method instead of one-on-one interviews to facilitate interactive dialogue and elicit a wide range of perspectives. Consistent with prior research by Coenen et al. [35] focus groups are recommended when the goal is to capture a broad view of participant perspectives. The group setting allowed participants to think about and build upon each other’s ideas, enabling a more in-depth understanding of common beliefs, barriers, and practices.

Surveys were administered prior to the focus groups to gather baseline information on participant demographics, sun protection behaviors, and current skin cancer prevention practices. This approach preserved the integrity of the quantitative data as it ensured that individual answers were not influenced by group discussion.

Combining survey data with qualitative insights from focus group discussions served to capture both individual-level behaviors and shared, community-level perspectives. Using both methods allowed us to connect what participants do with how they make sense of it, giving us a more complete understanding than either approach could on its own.

### Quantitative Data Analysis

We conducted descriptive analysis to analyze the sociodemographic and psychosocial factors using means for continuous variables and frequencies for categorical variables (SPSS software, version 29).

### Qualitative Data Analysis

Data analysis was conducted using rapid qualitative data analysis (RQA), a method comparable to traditional qualitative approaches and well-suited for structured, goal-driven studies with short time frames [36, 37]. Using recommended RQA practices [37, 38], the analysis followed a six-step process. In step 1, three members of the research team (DP, DSD, VG) independently reviewed a single focus group transcript and developed a neutral domain summary template based on focus group topics and questions (e.g. sun exposure at work, safety concerns and precautions, awareness of skin cancer risk factors, beliefs on skin cancer prevention and treatment). In step 2, the analysts met to discuss their summary templates and created a consolidated version of the summary template. In step 3, each analyst (DP, DSD, VG) independently summarized two focus group transcripts using the summary template. Each focus group was summarized by two analysts. In this summarization phase, the analysts developed descriptive summaries consisting of bullet statements organized by domains. Analysts also identified key quotes. In this phase, the goal was to develop a concise summary of the data avoiding data interpretation. In step 4, the analysts compared their focus group summaries and refined them through group discussion. Lastly, in step 5, the analysts compiled the focus group summaries into a matrix organized by domains. The team used the matrix to discuss patterns, connect domains, and identify themes.

## Results

### Sociodemographic Data

We conducted three focus groups with 25 participants. The first focus group consisted of nine participants, the second of six participants, and the third of ten participants. The majority of participants (88%) identified as male. The nationalities most represented among participants were Mexican (20%) and Salvadoran (20%). Approximately half of the focus group participants had less than a high school education. Income levels varied, with 32% earning between $10,000 and $39,999 annually. More than half of the participants (60%) lacked health insurance, while 36% were covered, predominantly through “Other” plans (16%). Employment status showed that 40% were employed full-time, 28% part-time, and 20% were unemployed. A detailed breakdown of all responses can be found in Table 1.

To reflect the mixed-methods nature of the study, the following results integrate quantitative survey findings with qualitative data from focus group discussions. Qualitative findings from focus groups are organized thematically, while quantitative survey data are embedded within each theme to provide additional context and support.

Analysis of the focus groups yielded four key themes: skin cancer awareness, skin cancer risk perception, skin cancer risk-reduction strategies, and barriers and facilitators for skin cancer risk-reduction behaviors.

### Skin Cancer Awareness

Participants had a low awareness of skin cancer. None agreed that it is one of the most common cancers globally, and there was low awareness regarding its presentation and symptoms. The word “cancer” itself was often associated with negative connotations and caused worry, leading some workers to dismiss the risks of skin cancer altogether. Participants indicated that the Hispanic community is not well-educated on skin cancer and has limited access to skin cancer information. As one participant expressed:

“Our community isn’t educated in this area of skin cancer. That’s why it’s bound to happen. Since we’re not educated on the topic, we don’t hear much about it. I say that local groups, like this, are very important in exposing and educating us because long-sleeved shirts, long pants, and hats can help us a lot, they can prevent something. Education is vital.”

(FG 1).

Most workers had no personal knowledge of anyone diagnosed with skin cancer, which contributed to a lack of awareness about the disease. Many expressed that in the Hispanic community, most people remain uninformed about conditions like skin cancer unless it directly affects them. Participants generally agreed that skin cancer can be treated, but they were unfamiliar with the types of available treatments. Many workers also reflected on how, even when wearing clothing, harmful UV rays can seep through and cause damage to the skin. While they understood that sun exposure could lead to skin cancer, some workers mentioned that extreme cold could also cause skin cancer.

### Skin Cancer Risk Perception

In the survey, 40% of participants reported not knowing how their risk compared to an average person, 28% believed they were less or equally likely to develop skin cancer and only 20% perceived themselves to be at higher risk. When asked about their future risk of developing skin cancer, 44% were neutral, 12% thought they were unlikely or very unlikely, and 32% believed they were likely to develop it. In terms of cancer worry, 28% never or rarely worried, 36% worried sometimes, and 20% worried often or always.

In the focus group discussions, many noted that people with darker skin tones were less prone to skin cancer, and some thought their skin could “get used to” sun exposure over time, building “resilience.” While some acknowledged the potential long-term effects of sun exposure, they often didn’t see themselves at risk unless they had experienced physical reactions like sunburns or heatstroke. Protection from the sun was believed to be most necessary from April through July.

Despite the severe consequences of sun exposure, such as headaches and heatstroke, participants often prioritized other more immediate hazards, such as machinery mishaps or falls, over long-term health issues like skin cancer. Reflecting this sentiment, one participant shared:

“Sun protection is not exactly one of the priorities in this type of work. There are many more immediate risks that are taken into consideration. Falling off a building is more immediate to consider than sun exposure. That is why it is left relegated, because there are things that are more immediate. And anything that takes time to appear will always end up being relegated.”

(FG 1).

This lack of awareness left workers with a minimal perception of their personal risk from sun exposure.

### Skin Cancer Risk-Reduction Strategies

In the survey, only about a third of the sample (32%) reported protecting their skin from the sun often or always, 28% reported protecting their skin sometimes while 28% reported rarely or never protecting their skin. When asked whether they use sun protection when they work outdoors for more than an hour, 56% of participants stated they do not. Among various types of sun protection, sunglasses were the most frequently used (57.1%), followed by long-sleeve shirts (52.4%), while the use of sunscreen was least reported (19.0%). A majority of participants (56.0%) reported seeking shade‘sometimes,’with 28% indicating they rarely or never seek shade (see Table 2).

Similarly, in the focus groups, participants mentioned that they commonly relied on clothing as their primary method of sun protection, frequently mentioning long sleeves, long pants, sunglasses, and hats. While many workers believed that protective clothing and sunglasses were effective in preventing skin cancer, there was a limited understanding of how different materials or colors could enhance protection. Sunscreen use was reported to be minimal, with some participants stating that they had never used it. This lack of use was in part explained by one participant, who remarked:

“You don’t put on sunscreen, thinking that cream is for women.”

(FG 3).

Based on additional focus group questions regarding risk-reduction strategies, workers were found to be generally aware that sunscreen could be beneficial but had limited knowledge of which to use and how to use them.

Per one of the participants:

“What we want, like they say, is to know more information about creams that can be used and all that, so we can take better care of our skin.”

(FG 3).

Additionally, there was not a clear understanding of the UV index within weather apps or knowledge of UV bracelets to indicate when sun protection was absolutely necessary.

“I didn’t know [about UV bracelets]. Where can we get them? That would be great.”

### Barriers and Facilitators for Skin Cancer Risk Reduction Behaviors

Participants reported varying levels of daily sun exposure, with 24% reporting working 6–9 h in the sun, and 16.0% spending 10 or more hours under direct sunlight. In the focus groups, participants also described long work hours, with some spending between ten to twelve hours per day in the sun.

#### Structural Barriers

Many participants mentioned the scarcity of shade, particularly in farm settings, where, in certain instances, there was no shade at all. Participants also described conditions where employers placed limitations on break periods and put productivity and profit above worker safety. As one participant expressed:

“The only thing they care about is getting it [work] done, no matter how we do it. They [employers] will always strive for productivity above all else.”

(FG 1).

Access to resources was also minimal. Most workers reported that their workplaces did not provide any information or guidance about skin cancer prevention. Workplace safety guidance primarily focused on immediate risks, such as preventing falls or addressing heatstroke, but did not include skin cancer education. One of the participants, in particular, stated:

“They [employers] don’t provide enough protective equipment, just shirts. They don’t mention the topic of cancer caused by the sun. I believe that in the talks they give, I have never heard that topic mentioned, which is why we are also so unaware.”

(FG 2).

Participants also highlighted barriers in access to health care services. As one participant explained:

“I have had a bit more complications because I didn’t have health insurance, I didn’t have a car, I didn’t have a license to get around, and I didn’t know English. For me, all of those were factors that prevented me from going to the doctor.”

(FG 2).

#### Subjective Barriers

Workers’ low use of sunscreen was further hindered by the discomfort of its “stickiness” while sweating during physical activity, a lack of knowledge about selecting effective products or understanding SPF levels, and minimal practice of skin self-examinations. As noted by one of the participants:

““It [wearing sunscreen] is uncomfortable when doing strenuous physical activity. If you’re sweating on top of it, it becomes a sticky mess on your face, besides the fact that you’re in a work environment where anything, dust and particles, all of that sticks to you. And that makes you feel very uncomfortable.”

(FG 1).

Barriers to self-exams included inconsistent practice, a lack of guidance or recommendations from healthcare providers, and uncertainty about what to look for during self-checks or when to seek medical care.

#### Cultural Barriers

Gender stereotypes, particularly those associated with “machismo,” discouraged many male participants from engaging in sun protection, as sunscreen use was often perceived as feminine.

According to a participant in particular:

“In the country where I come from, Mexico, people don’t use sunscreen because they think it’s vanity. They think it’s too vain, and if you’re a man, you’re being too vain.”

(FG 2).

Another factor contributing to low sun protection among participants was the perception of sunscreen as an “American” practice, unfamiliar or uncommon in participants’ countries of origin.

#### Facilitators

Despite the prevalence of these barriers, a limited number of facilitators emerged; some workplaces did provide protective clothing. When protective clothing was available, it was usually in the form of long-sleeve shirts with company logos. Larger companies, as opposed to smaller or more informal ones, were more likely to offer basic protection, such as sunscreen, long-sleeve uniforms, and hats. Other facilitators mentioned included a willingness to invest in UV-protective clothing, and the belief that encouragement from peers, family, and partners would play a significant role in promoting sunscreen use. To encourage sunscreen use, participants also emphasized the importance of using cultural role models, such as athletes, in modeling sun protective behaviors as well as clarifying misconceptions about skin cancer risks in darker-skinned individuals. A complete list of the barriers and facilitators that were identified across focus groups are listed in Table 3.

Table 4 presents a list of quotes that further highlight the themes discussed above.

## Discussion

This study aimed to examine the knowledge, risk perceptions, and behaviors related to sun exposure and skin cancer prevention among Hispanic outdoor workers, while identifying the specific barriers and facilitators for adopting sun protection practices. Recognizing that it may take a range of multi-level interventions to impact behavior, findings may be considered through the lens of individual, organizational, and societal influences.

### Individual-level

Findings from this study highlight significant gaps in both knowledge and perception of skin cancer risks, as well as insufficient sun protection behaviors. Our findings align with previous research that found suboptimal sun protection behaviors among Hispanic outdoor workers [23, 24, 26, 39], despite high exposure to UV radiation. For instance, in a study with male Hispanic outdoor day laborers, Niu and colleagues [23] found that more than half of Hispanic outdoor laborers reported experiencing at least one sunburn during the summer and that sun protection behaviors, such as wearing sunglasses, using sunscreen, or seeking shade, were generally low.

Our results underscore limited understanding of skin cancer prevention tools such as sunscreen, reflecting findings by prior studies [21, 31]. For example, in a qualitative study, Niu and colleagues [23] found that many participants reported a lack of knowledge about how to properly use sunscreen, including which SPF levels are most effective, the importance of reapplication, and the significance of the UV index. This lack of understanding may help explain similar findings by Day et al. [24], in which 69% of Hispanic outdoor workers said they rarely or never applied it while on the job. In light of this, interventions need to focus not just on making sunscreen available, but also on educating workers about proper use. Without this foundational knowledge, workers may not fully benefit from the protective properties of sunscreen.

Consistent with previous findings, this paper highlights how low knowledge and perceived risk continue to serve as key barriers to sun protection among Hispanic individuals.

### Organizational-level

Despite recognizing the effects of sun exposure, workers are more focused on immediate, physical risks like falls and heatstroke than on long-term health concerns such as skin cancer. Prolonged hours in direct sunlight, coupled with limited access to shade, create additional barriers to sun protection. This aligns with challenges seen in other studies that demonstrate how environmental and workplace conditions can limit the effectiveness of preventive measures, even when workers are aware of the risks [40].

In addition to environmental barriers, practical challenges around access to resources were identified. Participants reported that their workplaces offered limited protective resources and education regarding skin cancer prevention, with larger companies more likely to provide basic sun protection (e.g., protective clothing). This lack of institutional support for sun safety in smaller or informal work environments further exacerbates the health risks faced by Hispanic outdoor workers. The absence of workplace guidelines that specifically address skin cancer highlights a significant gap in occupational health and safety standards for this population. As identified by Walkosz et al. [41], employer-provided sun protection and training programs are essential for reducing risks in outdoor workers, but these initiatives remain underdeveloped in many industries, particularly in informal or small businesses, where Hispanics are more likely to be employed [42].

### Societal-level

The cultural barriers identified in this study align with those reported by prior that have found that cultural norms such as “machismo” commonly hinder sun protection among Hispanic individuals [18–24, 26, 31, 39, 43]. In our focus groups, similar attitudes were expressed, particularly around the perceived femininity of sunscreen use and the misconception that sun protection is unnecessary for darker skin tones. The concept of “machismo,” which emphasizes traditional masculine characteristics and the tendency to reject behaviors perceived as feminine (e.g., skin care), contributed to lower engagement in sun safety practices among male participants based on their shared perspectives [44]. This perception has been noted in prior studies, and our findings reinforce the need to address masculinity-related stigma when promoting sun safety in this population. Niu et al. [31] also highlighted facilitators such as family involvement, which align with our participants’ expressed openness to learning more when sun safety was framed as a way to protect loved ones. This reframing was especially powerful among male participants, some of whom initially expressed ambivalence toward their own risk. Shifting the focus from individual vulnerability to family well-being significantly increased participants’ engagement and openness to behavior change. This is especially meaningful within Hispanic communities where “familismo,” a cultural value emphasizing family interconnectedness and responsibility, plays a central role in decision-making [45]. By appealing to this collective sense of duty, sun safety messages could be more effective at motivating preventive action, especially among Hispanics that may otherwise minimize their personal risk or view sun protection as incompatible with traditional gender roles. This suggests that interventions grounded in culturally resonant values, such as protecting loved ones, may have greater impact than those centered solely on individual risk awareness.

Cultural factors, including the perception that sun protection measures like sunscreen are an American practice, also contribute to the limited use of preventive practices. As several studies have shown, culturally specific health beliefs and practices play a significant role in how individuals assess and manage health risks [46]. The low engagement in sun protection behaviors, such as sunscreen use, suggests that existing public health messages regarding skin cancer risk are not resonating with this community in a meaningful way.

### Recommendations

This study emphasizes the need for culturally and contextually appropriate interventions to reduce skin cancer risks among Hispanic outdoor workers.

Focus group findings revealed several facilitators that could be leveraged to improve sun protection among Hispanic outdoor workers. From a public health perspective, campaigns should address cultural beliefs and misconceptions surrounding sun exposure and skin cancer, ensuring that messages resonate with the target audience. These campaigns should integrate gender-specific considerations (e.g. addressing the stigmas around sunscreen use among men) and promote skin cancer awareness in a way that aligns with Hispanic cultural values. Participants emphasized the importance of using cultural role models in modeling sun protective behaviors and encouraging family involvement in sun protection practices.

Employers, particularly in small businesses and informal sectors, must play a more active role in protecting their workers. Policies that provide access to shade, regular breaks, and educational materials on skin cancer prevention (e.g. how to select effective sunscreen) could significantly mitigate the risks associated with prolonged sun exposure. Employers should also be encouraged to provide better access to protective clothing and sunscreen. Workers’ openness to using protective clothing and exploring other protection methods like sunscreen points to a critical opportunity for employers. Workplace policies and educational programs should moreover emphasize the importance of protection year-round, not just during high-risk months (April to August), especially given the long working hours and exposure experienced by many workers.

Healthcare providers also have a crucial role in educating this population about the risks of sun exposure and the importance of early detection through self skin examinations, which were of significant interest to participants. Training healthcare providers to effectively communicate with Hispanic populations, considering language barriers and cultural nuances, is critical in improving knowledge and prevention behaviors in this group. Additional barriers, such as insurance access and transportation, must also be prioritized. This could include supporting more safety net clinics, increased engagement in population health education and community events, advocating for expanded insurance coverage options, ensuring patients have the necessary support for telehealth services, and promoting transportation programs for underserved areas.

### Strengths and Limitations

The study has several strengths. Findings from this study contribute to the limited literature on skin cancer risk perception and sun protective behaviors barriers and facilitators in an understudied group of Hispanic outdoor workers from diverse industries. The mixed methods design constitutes another strength. The qualitative data allowed for the triangulation of the quantitative findings and provided an in-depth and nuanced understanding on outdoor workers’ awareness about skin cancer, skin cancer risk perception, and barriers and facilitators for engaging in protective behaviors. Findings from the study can inform the development of culturally-tailored interventions to reduce skin cancer disparities. Despite the strengths, several limitations should be considered when interpreting the results of this study. Our sample was mostly male participants, which can limit the generalizability of the findings to female outdoor workers. The study also focused on specific occupations (agriculture, landscaping, and construction), meaning the results may not fully reflect the experiences and behaviors of Hispanic outdoor workers in other industries. Another limitation is the reliance on self-reported data, which may have introduced social desirability bias or recall bias, potentially affecting the accuracy of participants’ responses regarding sun protection behaviors and their understanding of skin cancer. It is also important to recognize that the focus group format may have led to groupthink, where participants’ responses were influenced by others, potentially overlooking the views of those who were more reserved. Lastly, given our small sample size of 25 participants, future research should seek to include larger and more diverse samples that encompass a wider range of occupations to better capture the varied experiences of Hispanic outdoor workers. Additional studies should develop and refine culturally tailored interventions that directly address the knowledge gaps, facilitators, and barriers to sun protection identified by participants. Longitudinal research will be needed to assess behavior changes and evaluate the scalability and sustainability of culturally adapted interventions.

### Conclusion

The findings of this study underscore the complexity of skin cancer prevention within Hispanic outdoor worker populations, highlighting the interplay between cultural, social, and environmental factors. Although these workers face substantial sun exposure, their understanding of skin cancer and its risks remains limited, largely due to a combination of misinformation, structural barriers, and cultural factors. Improving skin cancer prevention among this population requires a multifaceted approach that integrates education, policy, and cultural considerations to ensure the adoption and maintenance of sun protective behaviors. Their willingness to learn about skin cancer and to embrace prevention strategies indicates that targeted interventions could be effective, but must be culturally sensitive and contextually appropriate to overcome the barriers that have been identified.

## Figures and Tables

**Table 1:** Sociodemographic Variables (*n* = 25)

Sociodemographic Variables	n(%)
**Gender**	
Male	22 (88.0%)
Female	3 (12.0%)
**Race**	
Black/African American	2 (8.0%)
White	3 (12.0%)
Multiracial	4 (16.0%)
Other	4 (16.0%)
Prefer not to respond	7 (28.0%)
**Hispanic/Latino**	
Yes	23 (92.0%)
No	0 (0%)
**Country of Birth**	
El Salvador	5 (20.0%)
Mexico	5 (20.0%)
Peru	4 (16.0%)
Colombia	2 (8.0%)
Nicaragua	2 (8.0%)
Venezuela	2 (8.0%)
Bolivia	1 (4.0%)
Dominican Republic	1 (4.0%)
Ecuador	1 (4.0%)
Guatemala	1 (4.0%)
**Marital Status**	
Divorced	1 (4.0%)
Married/Living with domestic partner	11 (44.0%)
Never married	9 (36.0%)
Prefer not to respond	3 (12.0%)
**Level of Education**	
Elementary school (through 5th grade)	2 (8.0%)
Middle school (6th-8th grade)	10 (40.0%)
High school diploma/GED	5 (20.0%)
Some college	3 (12.0%)
Bachelor’s degree	3 (12.0%)
**Annual income**	
Less than $10,000	5 (20.0%)
$10,000—$39,999	8 (32.0%)
$40,000—$79,999	3 (12.0%)
Prefer not to respond	5 (20.0%)
**Health insurance**	
No	15 (60.0%)
Yes	9 (36.0%)
**Type of insurance**	
Employer	2 (8.0%)
Medicare	3 (12.0%)
Private insurance	2 (8.0%)
Other	4 (16.0%)
**Current employment status**	
Full-time	10 (40.0%)
Part-time	7 (28.0%)
Unemployed	5 (20.0%)

**Table 2:** Sun exposure, skin protection, and skin cancer risk

Category	*N*(%)
**Daily hours working under sun**	
1–3 h	4 (16.0%)
3–6 h	7 (28.0%)
6–9 h	6 (24.0%)
10 h or more	4 (16.0%)
**When working outdoors for more than 1 hour on a sunny day, do you use sun protection?**	
I don’t know	1 (4.0%)
No	14 (56.0%)
Yes	7 (28.0%)
**How often do you protect your skin from the sun?**	
Never	5 (20.0%)
Rarely	2 (8.0%)
Sometimes	7 (28.0%)
Often	4 (16.0%)
Always	4 (16.0%)
**How often do you stay in the shade?**	
Never	3 (12.0%)
Rarely	4 (16.0%)
Sometimes	14 (56.0%)
Often	1 (4.0%)
**Types of sun protection used**	
Sunscreen	4 (19.0%)
Hat	10 (47.6%)
Sunglasses	12 (57.1%)
Long sleeve shirt	11 (52.4%)
Long pants	9 (42.9%)
Other	8 (38.1%)
**Compared to average person, chance of having skin cancer**	
More likely	5 (20.0%)
Equally likely	4 (16.0%)
Less likely	3 (12.0%)
I don’t know	10 (40.0%)
**How often are you worried about getting skin cancer?**	
Never	4 (16.0%)
Rarely	3 (12.0%)
Sometimes	9 (36.0%)
Often	2 (8.0%)
Always	3 (12.0%)
**What is your probability of getting skin cancer in the future?**	
Very unlikely	2 (8.0%)
Unlikely	1 (4.0%)
Neutral	11 (44.0%)
Likely	8 (32.0%)
**Have you been diagnosed with skin cancer?**	
No	23 (92.0%)
Yes	0 (0%)

**Table 3:** Participants’ perceptions of facilitators and barriers to sun protective practices

Behavior	Facilitators	Barriers
Seeking Shade	Potential for more regular use if shade is available and accessible Employer policies allowing for shade breaks could improve regular use	Shade is only used when available Difficulty finding shade in agricultural fields, minimal options in other industries Employer restrictions on shade breaks
Sun Protective Clothing	Clothing (hats, sunglasses, long sleeves, bandanas, and pants) is a practical and frequently used method of protection Awareness of the protective benefits of certain clothing materials and colors Willingness to invest in UV-protective clothing if available	Lack of availability or provision of protective clothing at work Workers often have to buy their own protective clothing Absence of industry standards for UV-protective clothing, leading to overpriced or misleading products Limited awareness of UV-protective clothing
Sunscreen Use	Encouragement from peers, family, and partners supports sunscreen use Increased education on SPF and sunscreen effectiveness Alluding to soccer athletes who are widely regarded as being “big, tall, and strong” to model the use of sunscreen among men and dispel gender stereotypes	Minimal use of sunscreen (typically applied once a day, if at all) Discomfort while sweating during physical activity (stickiness interferes with work) Gender stereotypes associating sunscreen use with femininity, leading to concerns about ridicule or bullying Lack of knowledge on how to select an effective sunscreen or understand SPF levels Cultural barriers (sunscreen seen as an“American”practice, not used in workers’countries of origin) Language barriers on product labels Fear of allergic reactions and the presence of a white cast in some sunscreens
Skin Self-Examination	Skin self-checks are easy to perform at home with no cost involved High motivation to learn how to perform self-checks effectively	Inconsistent practice of self-examinations Lack of guidance or recommendations from healthcare providers Uncertainty about what to look for during self-checks or when to seek medical care Many workers only visit the doctor when experiencing symptoms or issues due to concerns associated with cost, transportation, and language

**Table 4:** Participant quotes on skin cancer awareness, risk perception, risk-reduction strategies, and barriers and facilitators for risk-reduction behaviors

Theme	Quotes
Skin cancer awareness	[“Would you say skin cancer is something that worries the Latino community?”] “Of course.” “Honestly, the type of cancer I’ve heard the least about is skin cancer.” (FG 1) “I think we [Latinos] lack a lot of information about skin cancer; sometimes we don’t research these things. And we don’t start to inform ourselves until it is too late, once you are already sick. But if we did it earlier, we would have prevention, and we wouldn’t get sick.”(FG 2) “I am unaware of whether it is curable, what treatment it has; to be honest, I am completely unfamiliar with the topic. I know that skin cancer exists, but I don’t know about the treatment or how to prevent it.”(FG 2)
Risk perception	“In my opinion, I think skin cancer is one of the last concerns we have. In construction, I think people focus more on protecting themselves from dehydration or more severe physical injuries, like fractures and things like that. But skin cancer, I believe, is considered a last concern.”(FG 2) “No, I’m not really used to using those treatments [sunscreen], I have never applied them or used them, there has never been a need, thank God.”(FG 2) “When it comes to skin cancer, we mostly think about other diseases. We’re not worried about whether we’re going to get skin cancer or anything like that.” (FG 3)
Risk-reduction strategies	“What you could do to protect yourself was use gloves, a long-sleeved shirt, a hat, and then go under a tree when you had time.” (FG 2) “In construction, to protect yourself from the sun, obviously long sleeves, gloves…a balaclava to cover the face. And hats or caps, plus sunscreen.”(FG 2) “I used to work in landscaping. And there, everyone’s out in the sun. It’s rare that you get to work in the shade. You work constantly, like three or four hours, then you take a break and go under shade, somewhere nearby.”(FG 2)
Barriers and facilitators for risk-reduction behaviors	“For reasons of comfort and practicality, the most chosen protection is clothing.” (FG 1) [Do you think it would be a barrier or an inconvenience to pay a little more if you knew that the clothing had UPF specifications?] “No, not for me.” “If you give a 100% guarantee [that the clothing has UPF], we will be happy to do it.” (FG 1) “I just started working in roofing when I arrived. It is a job that puts you in the sun all day long. There is also the habit of not using sunscreen, no moisturizer for your skin. So you are 100% exposed to the sun, with sunburns on your face, arms, and neck.” (FG 2) “Going to a doctor here is not easy; they charge a lot of money, which is why we prefer to endure it sometimes. I think about taking the last pill I can find that works before going to a hospital because it is very expensive.”(FG 2) “I think I started using sunscreen when I was fifteen or sixteen. I didn’t like it because I started sweating really badly.” (FG 3) [“How could we take away the taboo that creams are for women?”] “Using soccer players on the field, they’re tough men and can be used for publicity. They’re big, tall, and strong.” (FG 3) [“If companies had sunscreen available, would you use them?”] “It would be helpful, but I think it would depend on the education the person has.” (FG 3)

## Data Availability

The authors welcome inquiries from investigators interested in possible collaboration and use of de-identified data from this study. The data has not been placed into a public repository.
