# Correction: Exposing the Risk: Identifying Perspectives on Skin Cancer Awareness and Prevention Opportunities Among Hispanic Outdoor Workers

**DOI:** 10.1007/s40615-025-02692-3

**Published:** 2025-10-20

**Authors:** David Perez, Dariana Sedeño-Delgado, Carlos Orellana García, Valeria Gómez, Chiranjeev Dash, Roxanne Mirabal-Beltran, Alejandra Hurtado-de-Mendoza

**Affiliations:** 1 Georgetown University Medical Center, Washington, DC USA; 2 Baylor College of Medicine, Houston, TX USA; 3 Georgetown University Berkley School of Nursing, Washington, DC USA; 4 Jess and Mildred Fisher Center for Hereditary and Clinical Cancer Genomics Research, Washington, DC USA; 5 Lombardi Comprehensive Cancer Center, Georgetown University, 2115 Wisconsin AVE NW, Suite 300, Washington, DC 2000 USA

**Correction: Journal of Racial and Ethnic Health Disparities**

**https://doi.org/10.1007/s40615-025-02623-2**

The article “Exposing the Risk: Identifying Perspectives on Skin Cancer Awareness and Prevention Opportunities Among Hispanic Outdoor Workers”, written by David Perez et al. was originally published electronically on the publisher’s internet portal on 5 September 2025 without open access. With the author(s)’ decision to opt for open access, the copyright of the article changed on 26 September 2025 to © The Author(s) 2025 and the article is forthwith distributed under a Creative Commons Attribution 4.0 International License, which permits use, sharing, adaptation, distribution and reproduction in any medium or format, as long as you give appropriate credit to the original author(s) and the source, provide a link to the Creative Commons license, and indicate if changes were made. The images or other third party material in this article are included in the article's Creative Commons license, unless indicated otherwise in a credit line to the material. If material is not included in the article's Creative Commons license and your intended use is not permitted by statutory regulation or exceeds the permitted use, you will need to obtain permission directly from the copyright holder. To view a copy of this license, visit http://creativecommons.org/licenses/by/4.0/.

